# Inhibition of connexin 43 attenuates oxidative stress and apoptosis in human umbilical vein endothelial cells

**DOI:** 10.1186/s12890-019-1036-y

**Published:** 2020-01-21

**Authors:** Jia-wei Ma, Dan-dan Ji, Qian-qian Li, Ting Zhang, Liang Luo

**Affiliations:** 1 Department of Critical Care Medicine, The Affiliated Wuxi No.2 People’s Hospital of Nanjing Medical University, Wuxi, 214002 China; 2 Department of Clinical Laboratory, The Affiliated Wuxi Matemity and Child Health Care Hospital of Nanjing Medical University, Wuxi, 214002 China

**Keywords:** Acute respiratory distress syndrome, Endothelial cell, Mitochondrion, Connexin 43, Lipopolysaccharides, Oxidative stress, Apoptosis

## Abstract

**Background:**

Previous studies demonstrated an important role for connexin 43 (Cx43) in the regulation of apoptosis by influencing mitochondrial functions. This study aimed to investigate the relationship between Cx43 and lipopolysaccharide (LPS)-induced oxidative stress and apoptosis in human umbilical vein endothelial cells (HUVECs).

**Methods:**

Western blot was performed to determine mitochondrial Cx43 (MtCx43) protein level and phosphorylation (p-MtCx43). Gap19, a selective Cx43 inhibitor, was used to examine the effects of Cx43 on LPS-induced oxidative stress and apoptosis in HUVECs. Expression of regulatory genes associated with oxidative stress was examined by quantitative polymerase chain reaction (qPCR) and Western blot. Apoptosis was assessed by flow cytometry.

**Results:**

LPS stimulation resulted in increased levels of MtCx43 and p-MtCx43. Interestingly, Gap19 antagonized the upregulation of glutathione S-transferase Zeta 1 (GSTZ1) and cytochrome b alpha beta (CYBB), and the downregulation of antioxidant 1 (ATOX1), glutathione synthetase (GSS) and heme oxygenase 1 (HMOX1) induced by LPS or Cx43 overexpression. Moreover, the increased production of reactive oxygen species (ROS) and apoptosis elicited by LPS or Cx43 overexpression were reduced following treatment with Gap19.

**Conclusions:**

Selective inhibition of Cx43 hemichannels protects HUVECs from LPS-induced apoptosis and this may be via a reduction in oxidative stress production.

## Background

Acute respiratory distress syndrome (ARDS) is characterized by severe pulmonary inflammation, increased capillary endothelial permeability and a high mortality rate [1, 2]. Lipopolysaccharide (LPS), a bacterial endotoxin and potent mediator of endothelial activation, induces pro-inflammatory cytokines and adhesion molecules, as well as the generation of reactive oxygen species (ROS), oxidative stress, apoptosis, inflammation, pulmonary vascular endothelial cell dysfunction, and pulmonary microvascular permeability [3–5]. LPS-induced dysfunction of pulmonary vascular endothelial cells is clinically important as it presents early in the course of ARDS and is associated with higher mortality [6, 7]. However, the molecular mechanisms remain elusive.

LPS-induced mitochondrial dysfunction plays an important role in the induction of apoptosis [8, 9]. LPS initiates oxidative stress, which could trigger the opening of the high-conductance mitochondrial permeability transition pore in mitochondrial membranes, resulting in proton leak [10, 11]. Mitochondrial permeability transition has been associated with matrix swelling, unwinding of respiratory chain, Ca^2+^ efflux, loss of membrane potential, overproduction of ROS and release of cytochrome c, ultimately leading to apoptosis. Connexin 43 (Cx43), which is commonly found in the plasma membrane where it forms gap junction channels and facilitates intercellular communication, is also present in mitochondrial membranes as hemichannels of various cell types [12]. Studies in cardiomyocytes and retinal endothelial cells have shown that mitochondrial Cx43 (MtCx43) acts as an important regulator of apoptosis by influencing mitochondrial respiration, matrix ion fluxes and ROS production [13–15]. However, the role of Cx43 in pulmonary vascular endothelial cells is not well understood. Specifically, the effects of LPS on Cx43 expression in the mitochondria in pulmonary vascular endothelial cells remains unclear, and whether Cx43 expression and channel activity play critical roles in oxidative stress and apoptosis has yet to be established.

Preventing accelerated apoptosis of pulmonary microvascular endothelial cells (PMVEC) is an important treatment endpoint in ARDS. Thus, identifying novel molecular players regulating oxidative stress and apoptosis could provide new insights into understanding how LPS induces injury in the pulmonary vasculature. In the current study, we examined the effects of LPS on MtCx43 expression, as well as the impact of Cx43 inhibition on LPS-induced oxidative stress and apoptosis in human umbilical vein endothelial cells (HUVECs). Our results showed that LPS stimulation resulted in elevated expression of Cx43 and induction of oxidative stress and apoptosis in HUVECs. Such effects of LPS were reduced upon selective inhibition of Cx43 by Gap19. Taken together, these results suggest that Cx43 may be involved in mediating LPS-induced oxidative stress and apoptosis in HUVECs.

## Materials and methods

### Reagents

HUVECs were obtained from ScienCell Research Laboratories (San Diego, CA, USA). Dulbecco’s modified Eagle’s medium (DMEM) and 10% fetal bovine serum (FBS) were obtained from GIBCO (Grand Island, NY, USA). LPS was obtained from Sigma-Aldrich (St. Louis, MO, USA). Fluorescein isothiocyanate (FITC)-labeled annexin V (Annexin V-FITC) Apoptosis Detection Kit (C1062) containing binding buffer was obtained from Beyotime Biotechnology (Shanghai, China). Mitochondrial isolation kit was purchased from Thermo Fisher Scientific (Paisley, UK). Gap19 was obtained from Bio-Techne (Tocris Bioscience, Sussex, UK). Lipofectamine 2000 and TRIzol were obtained from Invitrogen (Carlsbad, CA, USA). cDNA Archive Kit was purchased from Applied Biosystems (Foster City, CA, USA). SYBR Green was obtained from Takara (Otsu, Shiga, Japan). RNase Inactivation Reagent was obtained from Thermo Fisher Scientific (Waltham, MA, US). Bicinchoninic acid (BCA) protein assay kit was purchased from Pierce Chemical Co. (Rockford, IL, USA).

### Cell culture

HUVECs were cultured in DMEM containing 10% FBS at 37 °C and 5% CO_2_. Cells in the logarithmic growth phase were seeded in culture plates and allowed to grow to confluence.

### Generation of constructs for stable transfection

To overexpression of Cx43, Cx43 coding sequence was cloned into pLKO.1 vector using *Age*l I and *Eco*l I. Blank pLKO.1 vector was used as a negative control (NC). Constructs containing 1 μg pLKO.1-Cx43, 0.9 μg psPAX2 and 0.1 μg pMD2G were then co-transfected into HEK 293 T cells at 80–90% confluence using Lipofectamine 2000 according to the manufacturer’s instruction. After incubation in a CO_2_ incubator at 37 °C, vector was collected 48 h after transfection and used to transduce HUVECs. Blank pLKO.1 vector used as negative control.

### Experimental groups

Cells were treated as follows: Experiment 1, HUVECs were treated with varying concentrations of LPS in 1% FBS and cultured for 24 h to induce injury, and HUVECs without treatment were used as control; Experiment 2, HUVECs were randomly divided into different groups: 100 μM Gap19 treatment group, 400 ng/mL LPS treatment group, 400 ng/mL LPS combined with 100 μM Gap19 treatment group, and sterile double distilled water or dimethyl sulfoxide (DMSO) alone was used as controls; Experiment 3, HUVECs were randomly divided into different groups: blank pLKO.1 vector transduction group, pLKO.1-Cx43 vector transduction group, pLKO.1-Cx43 transduction combined with 100 μM Gap19 treatment group, and untreated HUVECs were used as control. HUVECs were treated with LPS and/or Gap19 both for 24 h.

### Apoptosis analysis

HUVECs in logarithmic growth phase were plated in 6-well plates at a density of 3 × 10^5^ cells/well. After treatment, HUVECs were digested with trypsin containing ethylenediaminetetraacetic acid (EDTA), washed twice with phosphate buffered saline (PBS) and centrifuged at 1000 g for 5 min. The cell pellet was resuspended in binding buffer and apoptotic rate was examined using Annexin V-FITC Apoptosis Detection Kit according to the manufacturer’s instructions. Apoptotic cells were quantified using a flow cytometer (excitation wavelength 488 nm, emission wavelength 535 nm; BD Accuri C6, software version 1.0.264.21; BD Biosciences, Franklin Lakes, NJ, USA). The data were analyzed using FlowJo software (FlowJo, Ashland, OR, USA). Apoptotic cells were defined by early apoptosis (Annexin V-positive/PI-negative) and late apoptosis (Annexin V-positive/PI-positive).

### Measurement of intracellular ROS

HUVECs in logarithmic growth phase were plated at a density of 5 × 10^5^ cells/well in 6-well plates. Following treatment, HUVECs were washed twice with PBS and digested with trypsin and then centrifugated at 1000 g for 5 min. Cells were collected and resuspended at a density of 1 × 10^6^ cells in 1 mL PBS and 10 μM 2′, 7′-Dichlorodihydrofluorescein diacetate (DCFH-DA), and then incubated for 20 min at 37 °C in the dark, followed by subsequent washes (3x) with serum-free media. Fluorescence was analyzed at 485 nm (excitation) and 535 nm (emission) using a flow cytometer (BD Accuri C6).

### Real-time reverse transcription-PCR

Total RNA was extracted from cell lines using TRIzol and stored at − 80 °C in RNase Inactivation Reagent. A total of 1 μg of RNA was reverse transcribed using the High Capacity cDNA Archive Kit. Real-time PCR was carried out using SYBR Green and performed on the GeneAmp PCR Systems 2700 (Applied Biosystems) according to the manufacturer’s protocol. Primers were designed by the Primer Express software and were listed as followed: Glutathione S-transferase Zeta 1 (GSTZ1), Forward: 5′-GCCCAGAACGCCATCACTT-3′, Reverse: 5′-CTACACAGTATATGCCCGCTG-3′; Cytochrome b alpha beta (CYBB), Forward: 5′-ACCGGGTTTATGATATTCCACCT-3′, Reverse: 5′-GATTTCGACAGACTGGCAAGA-3′; Antioxidant 1 (ATOX1), Forward: 5′-GTGCTGAAGCTGTCTCTCGG-3′, Reverse: 5′-GCCCAAGGTAGGAAACAGTCTTT-3′; Glutathione synthetase (GSS), Forward: 5′-GGGAGCCTCTTGCAGGATAAA-3′, Reverse: 5′-GAATGGGGCATAGCTCACCAC-3′; Heme oxygenase 1 (HMOX1), Forward: 5′-AAGACTGCGTTCCTGCTCAAC-3′, Reverse: 5′-AAAGCCCTACAGCAACTGTCG-3′; Glyceraldehyde-3-phosphate dehydrogenase (GAPDH), Forward: 5′-ACAACTTTGGTATCGTGGAAGG-3′, Reverse: 5′-GCCATCACGCCACAGTTTC-3′. Expression levels are given as ratios to GAPDH.

### Cell surface biotinylation

To examine the Cx43 expression in plasma membrane of HUVECs, HUVECs were seeded in 100-mm dishes and treated without or with different concentrations of LPS. After treatment, cells were biotinylated with EZ-link Sulfo-NHS-SS-Biotin (Pierce Biotechnology) as previously described [16], after which cell lysates were collected and subjected to SDS-PAGE electrophoresis and Western blotting (see below).

### Western blot analysis

Cells were lysed in radio immunoprecipitation assay (RIPA) lysis buffer. Extracts of mitochondrial fractions were prepared using the Qproteome Mitochondria Isolation Kit (Qiagen) according to the manufacturer’s instructions. Protein concentration was detected by BCA assay. Then, 15 μL proteins were resolved on 12% sodium dodecyl sulfate-polyacrylamide gel electrophoresis (SDS-PAGE) gels and transferred to polyvinylidene fluoride (PVDF) membrane. Membranes were then blocked with 5% free-fat milk for 1 h at room temperature and incubated with primary anti-phospho-Cx43, anti-Cx43, anti-COX IV, anti-GSTZ1, anti-CYBB, anti-ATOX1, anti-GSS, anti-HMOX1, and anti-GAPDH antibodies for 2 h at room temperature. Subsequently, membranes were incubated with horseradish peroxidase (HRP)-labeled Goat Anti-Rat IgG for 1 h at 37 °C, followed by washing 3 times with Tween-20 (TBST) and then developed using an enhanced chemiluminescence detection kit. Intensity of the bands was detected and analyzed using Quantity One analyzing system (Bio-Rad Laboratories, Inc., Hercules, CA, USA).

### Statistical analysis

Continuous variables were presented as mean ± standard deviation (SD). Statistical analyses were performed in GraphPad Prism (Version 5, GraphPad Software, Inc., San Diego, CA). Statistical analyses were assessed by One-way analysis of variance, followed by Tukey’s post hoc test, and probability (p) values less than 0.05 were considered statistically significant.

## Results

### LPS elevates the expression of Cx43 in mitochondria and plasma membrane of HUVECs

**Fig. 1:**
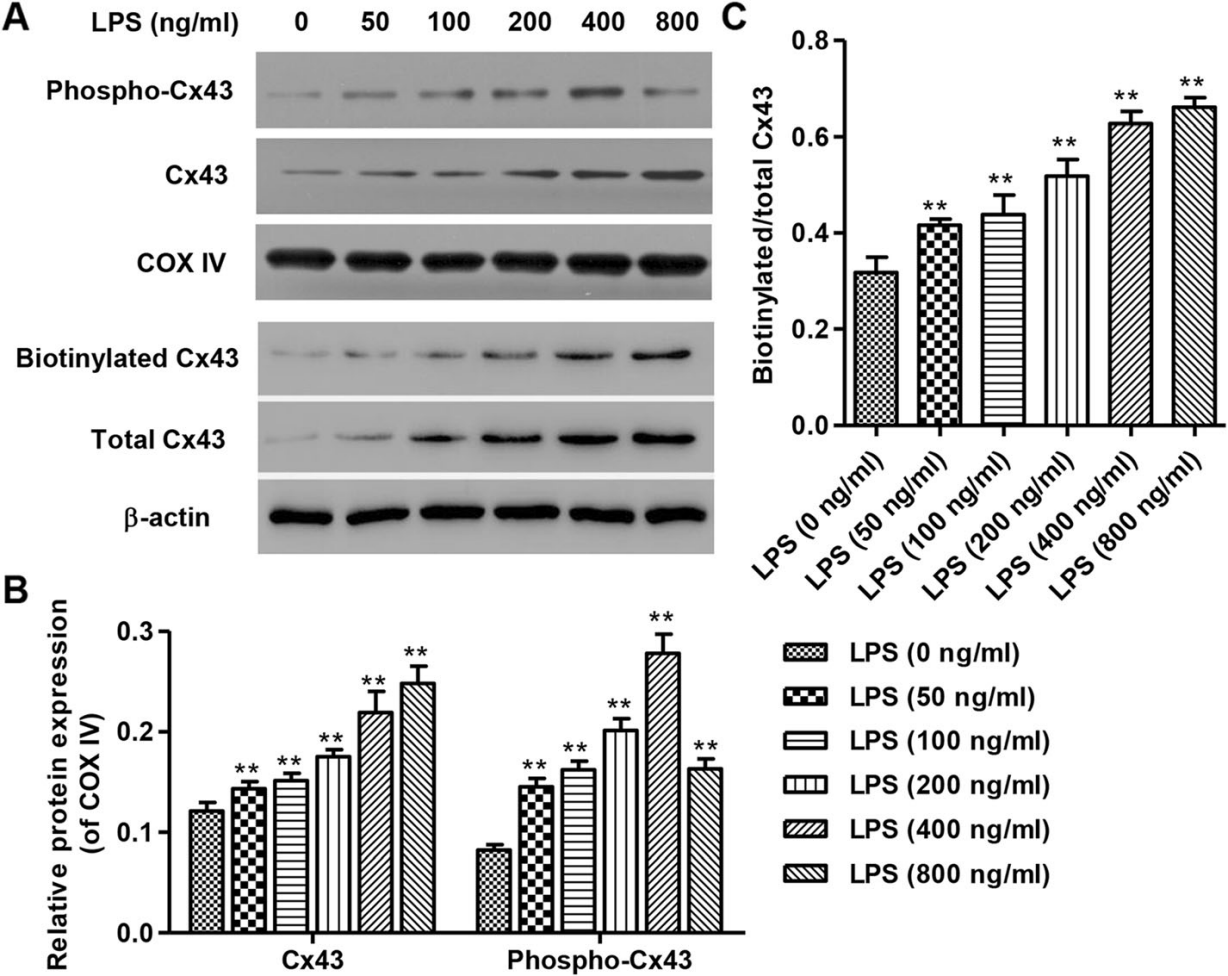
Expression of Cx43 and phospho-Cx43 after 24 h LPS stimulation. After continuous exposure to LPS for 24 h, protein levels of Cx43 in mitochondria and plasma membrane in HUVECs were detected by Western Blot (**a**), which showed concentration-dependent effects from 0 to 800 ng/mL, with 800 ng/mL LPS stimulation achieving the strongest expression of Cx43 (**b**, **c**). Biotinylated Cx43 is a representation of plasma membrane Cx43. Expression of mitochondrial phospho-Cx43 was detected by Western Blot (**a**), which likewise showed concentration-dependence effects from 0 to 400 ng/mL, with 400 ng/mL LPS stimulation achieving the strongest expression of phospho-Cx43 (**b**). COXIV and β-actin were used as loading control. Experiments were performed in 3 biological replicates and data are presented as mean ± SD. **P < 0.01 compared with 0 ng/ml LPS

Continuous exposure to LPS for 24 h resulted in increased expression of Cx43 in mitochondria and plasma membrane of HUVECs, as shown by Western blot (Fig. 1a). Biotinylated Cx43 is a representation of plasma membrane Cx43. This occurred in a concentration-dependent manner, with 800 ng/mL LPS stimulation achieving the strongest expression of Cx43 (Fig. 1b and c). Similarly, phospho-Cx43 expression in the mitochondria exhibited concentration-dependent effects from 0 to 400 ng/mL (Fig. 1a), with 400 ng/mL LPS stimulation achieving the strongest expression of phospho-Cx43 (Fig. 1b).

### LPS induces apoptosis in HUVECs

**Fig. 2:**
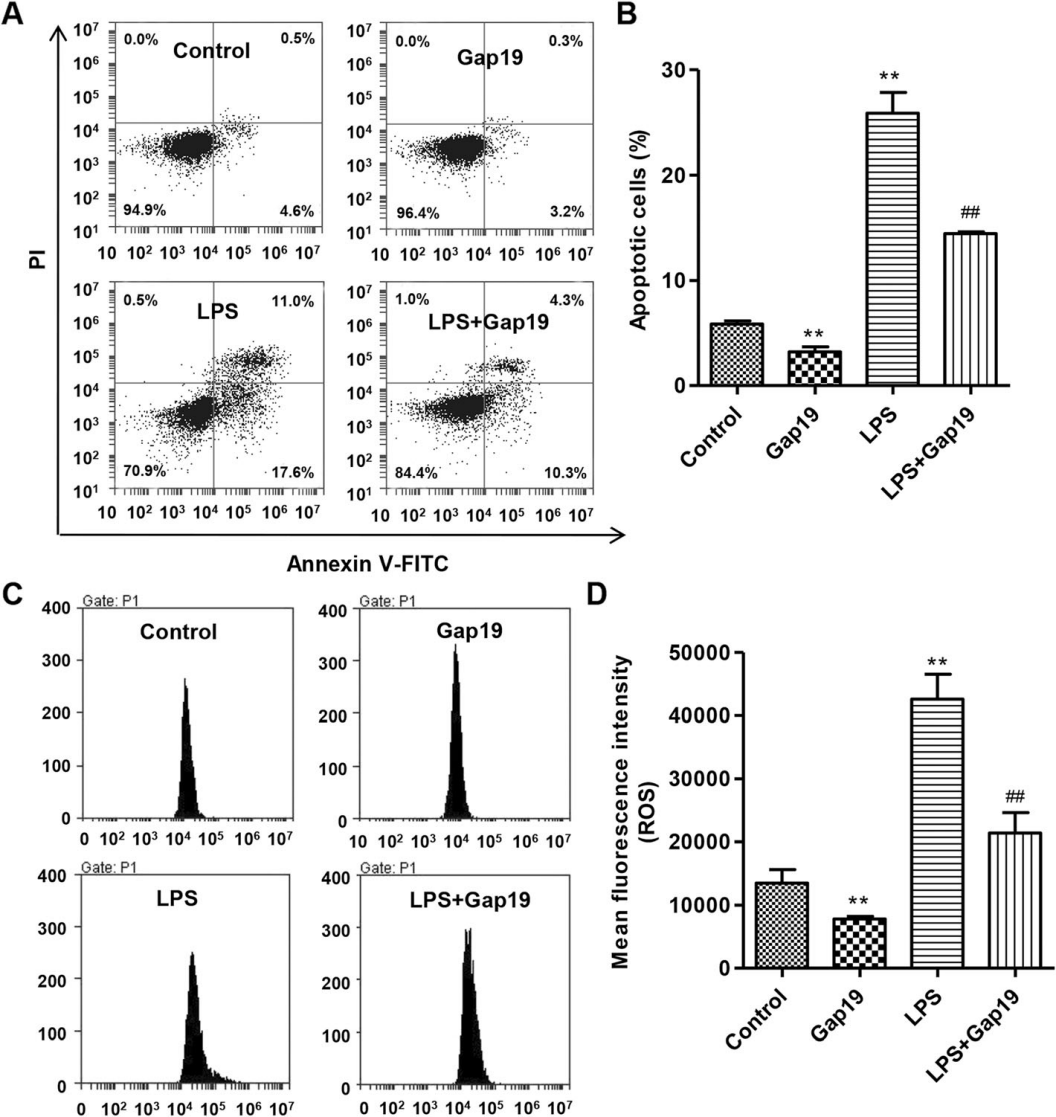
Effects of LPS stimulation with or without Gap19 treatment on apoptosis and ROS levels in HUVEC. After continuous exposure to 400 ng/mL LPS with or without 100 μM Gap19 for 24 h, apoptosis (**a**, **b**) and ROS levels (**c**, **d**) in HUVECs were assessed, respectively, by Annexin V-FITC/PI and DCFH-DA using flow cytometry. Gap19 was used as an inhibitor of Cx43. Experiments were performed in 3 biological replicates and data are presented as mean ± SD. **P < 0.01 compared with control. ^##^*P* < 0.01 compared with LPS

Flow cytometry analysis was conducted to examine the effects of LPS on apoptosis in HUVECs. As shown in Fig. 2a and b, exposure to 400 ng/mL LPS for 24 h increased apoptosis by 4.43-fold compared with control HUVECs, which was markedly reduced by 44.3% after Gap19 treatment. Moreover, treatment with Gap19 alone reduced apoptosis by 45.2% compared with control HUVECs, suggesting that Gap19 reduced apoptosis in an LPS-independent manner.

### LPS increases intracellular ROS production in HUVECs

Intracellular ROS levels were measured in HUVECs by flow cytometry. As shown in Fig. 2c and d, LPS dramatically increased intracellular ROS levels by 3.15-fold compared with control HUVECs, which were markedly reduced by 49.8% after Gap19 treatment. Treatment with Gap19 alone reduced intracellular ROS levels by 42.1% compared with control HUVECs, suggesting that Gap19 reduced intracellular ROS production in an LPS-independent manner.

### Effects of LPS on the expression of regulatory genes related to oxidative stress in HUVECs

**Fig. 3:**
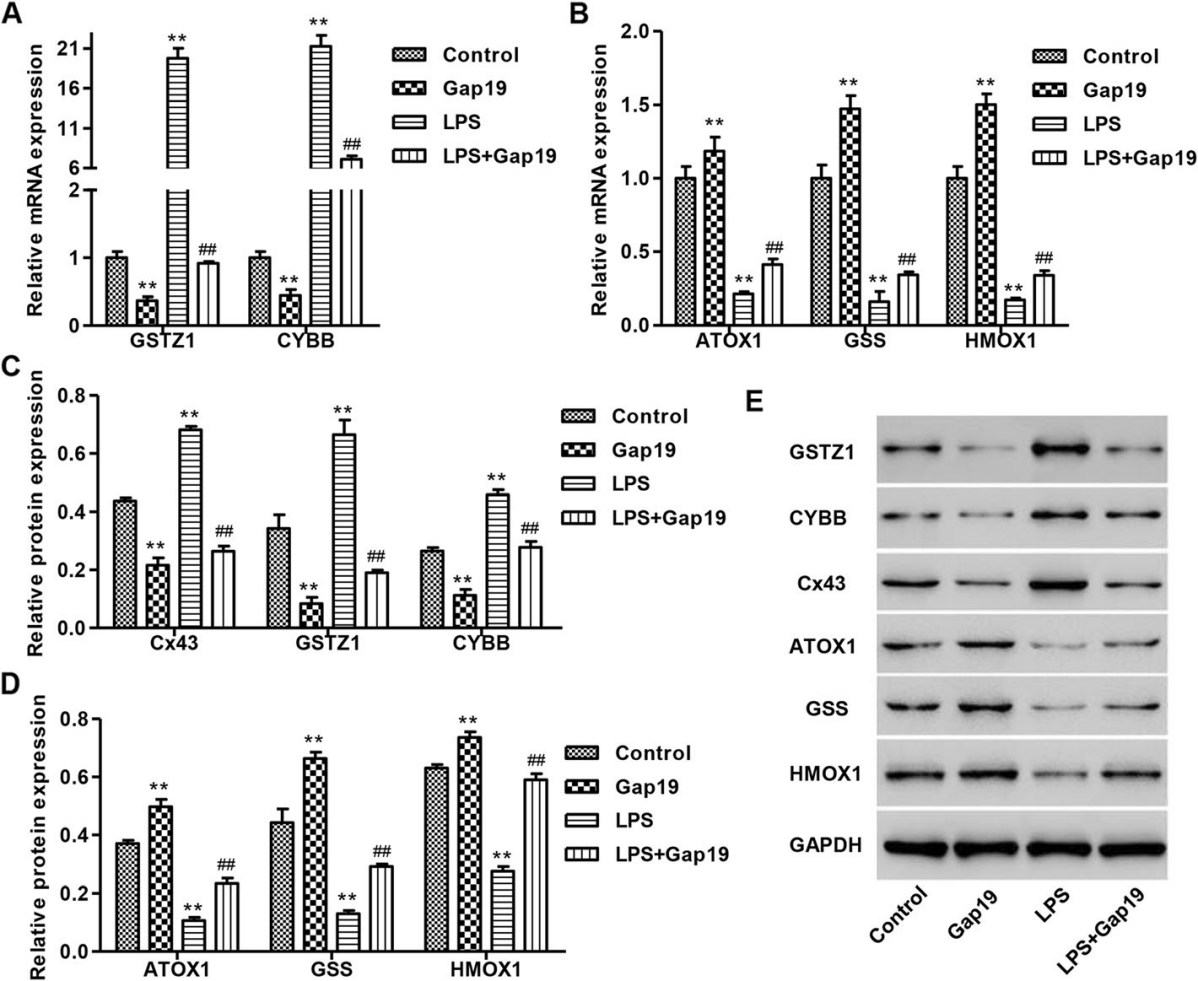
Effects of LPS stimulation on the expression of regulatory genes related to oxidative stress in HUVECs. After continuous exposure to 400 ng/mL LPS with or without 100 μM Gap19 for 24 h, mRNA expression and protein levels of regulatory genes related to oxidative stress were determined by qPCR (**a**, **b**) and Western blot (**c**-**e**), respectively. GAPDH was used as a loading control. Experiments were performed in 3 biological replicates and data are presented as mean ± SD. **P < 0.01 compared with control. ^##^*P* < 0.01 compared with LPS

GSS, GSTZ1, ATOX1 and HMOX1 are antioxidant factors that play important roles in oxidative stress resistance [17–20]. CYBB is a subunit of NADPH oxidase, which is involved in the production of ROS, such as superoxide and hydrogen peroxide. After continuous exposure to 400 ng/mL LPS for 24 h in HUVECs, the mRNA expression of these genes was examined by real-time PCR. As shown in Fig. 3a, LPS treatment elevated the mRNA expression of GSTZ1 and CYBB by 19.8-fold and 21.3-fold, respectively. Gap19 alone reduced the mRNA expression of GSTZ1 and CYBB. Moreover, Gap19 treatment blocked LPS-induced increases in mRNA expression of GSTZ1 and CYBB by 95.4 and 66.5%, respectively.

The mRNA expression of ATOX1, GSS and HMOX1 in HUVECs was also examined. As shown in Fig. 3b, LPS treatment reduced the mRNA expression of ATOX1, GSS, and HMOX1 by 78.8, 83.7, and 82.5%, respectively. Treatment with Gap19 alone in HUVECs increased ATOX1, GSS and HMOX1 mRNAs. Moreover, Gap19 treatment blocked LPS-induced decreases in the mRNA expression of ATOX1, GSS, and HMOX1, respectively. Similar results were observed in the protein expression of GSTZ1, CYBB, ATOX1, GSS, and HMOX1 in HUVECs as shown by Western blot (Fig. 3c-e).

### Gap19 suppresses apoptosis induced by Cx43 overexpression in HUVECs

**Fig. 4:**
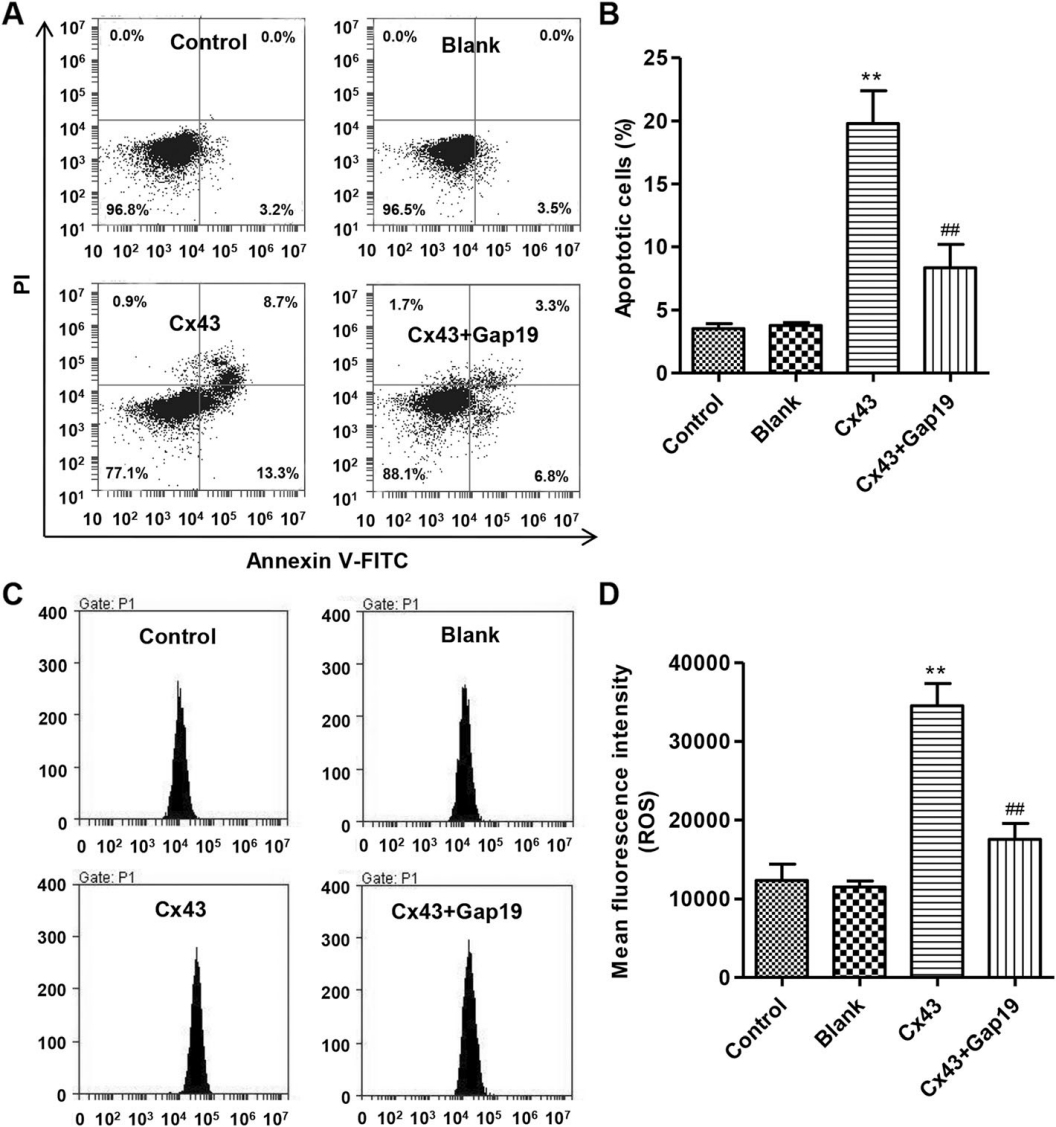
Effects of Cx43 overexpression and Gap19 treatment on apoptosis and ROS production in HUVECs. After Cx43 overexpression with or without 100 μM Gap19 for 24 h, apoptosis and ROS production were assessed, respectively, by Annexin V-FITC/PI and DCFH-DA using flow cytometry. Cx43 overexpression and Gap19 treatment showed decreased cell apoptosis (**a**, **b**) and ROS production (**c**, **d**) in HUVECs. Experiments were performed in 3 biological replicates and data are presented as mean ± SD. **P < 0.01 compared with control. ^##^*P* < 0.01 compared with Cx43 overexpression

After overexpression of Cx43 with or without Gap19 treatment in HUVECs, apoptosis was assessed by flow cytometry. As shown in Fig. 4a and b, overexpression of Cx43 markedly increased apoptosis by 7.11-fold compared with control, while Gap19 treatment blocked Cx43 overexpression-induced apoptosis by 47.2%.

### Gap19 decreases intracellular ROS levels induced by Cx43 overexpression in HUVECs

Flow cytometry was conducted to examine the effects of Cx43 overexpression with or without Gap19 treatment on intracellular ROS levels in HUVECs. As shown in Fig. 4c and d, overexpression of Cx43 increased intracellular ROS levels by 2.80-fold compared with control HUVECs, while Gap19 treatment markedly suppressed intracellular ROS levels induced by Cx43 overexpression by 49.3%.

### Effects of Gap19 on Cx43 overexpression-induced changes in oxidative stress-related genes in HUVECs

**Fig. 5:**
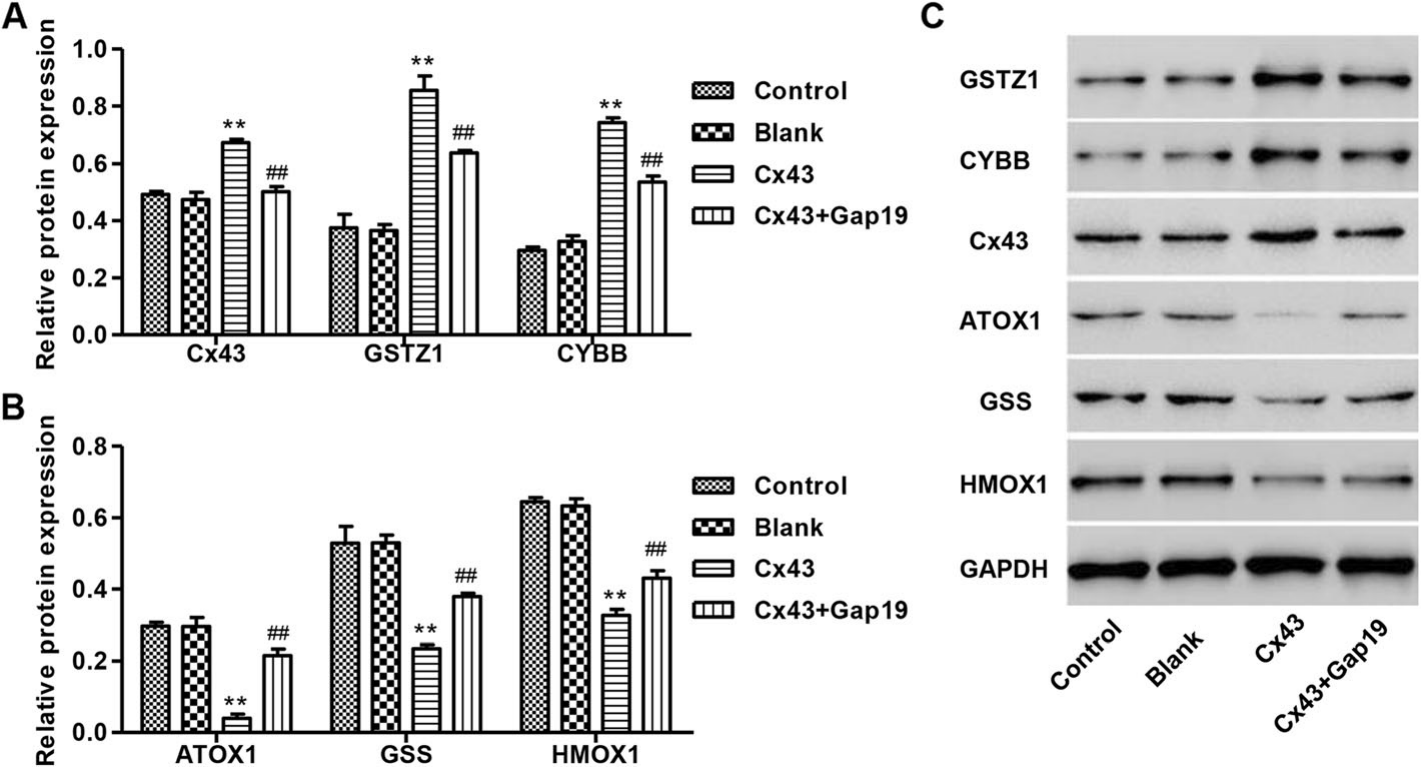
Expression of regulatory genes related to oxidative stress in HUVEC after Cx43 overexpression with or without Gap19 treatment. **a**-**c** After Cx43 overexpression with or without 100 μM Gap19 for 24 h, protein levels of regulatory genes related to oxidative stress were detected by Western blot. GAPDH was used as a loading control. Experiments were performed in 3 biological replicates and data are presented as mean ± SD. **P < 0.01 compared with control. ^##^*P* < 0.01 compared with Cx43 overexpression

Following overexpression of Cx43 with or without Gap19 treatment in HUVECs, protein expression of GSTZ1, CYBB, ATOX1, GSS and HMOX1 was determined by Western blot. Our results showed that Cx43 overexpression increased the protein levels of Cx43, GSTZ1, and CYBB by 37, 128, and 151% compared with control, respectively. These effects were markedly reduced after Gap19 treatment (Fig. 5a and c). Moreover, Cx43 overexpression decreased the protein levels of ATOX1, GSS, and HMOX1 by 86.7, 55.7, and 49.3% compared with control, respectively, and these effects were reversed by Gap19 treatment (Fig. 5b-c).

## Discussion

This study demonstrated that LPS (from 50 to 400 ng/mL) upregulated MtCx43 expression and phosphorylation in a concentration-dependent manner. In addition, 400 ng/mL LPS treatment induced oxidative stress and apoptosis in HUVECs. The effects of LPS and Cx43 overexpression on oxidative stress and apoptosis were reduced by Gap19, a nonapeptide derived from the cytoplasmic loop (CL) of Cx43 as a hemichannel blocker [15]. Taken together, these two findings suggest an involvement of Cx43 in LPS-mediated oxidative stress and apoptosis in HUVECs.

As exposure of endothelial cells to LPS plays a central role in the pathogenesis of ARDS [21, 22], our cell culture model using LPS-induced HUVECs to investigate the influence of ARDS on oxidative stress and apoptosis may provide helpful insights into understanding ARDS pathogenesis, despite that HUVECs may not completely recapitulate the pulmonary disorder in ARDS due to the complexity of the disease. Compared with HUVECs, other endothelial cells such as arterial, venous, arteriolar, venular and capillary ECs may be significantly heterogeneous and contribute differently to LPS-induced pathogenesis of ARDS. Therefore, more sophisticated experimental research in the future is important.

It has been shown that Cx43 hemichannels cause cell death of renal epithelial cells [23], while Cx43 gap junction channels confer protection to human retinal pigment epithelial cell line against cell death [24]. In the present study, LPS stimulation resulted in oxidative stress and elevation of Cx43 expression in plasma membrane and whole-cell lysate, which was in line with the previous study showing that oxidative stress increased surface expression of Cx43 in osteocyte cell line, and that Cx43 knockdown blocked apoptosis induced by oxidative stress [16]. However, Smyth et al. reported that oxidative stress decreased surface expression of Cx43 in mouse ventricular cardiomyocytes, perturbing Cx43 forward trafficking [25]. These data indicate that changes in Cx43 occurring in different cell compartments may be responsible for the various functions. Cx43 phosphorylation can be mechanistically linked to changes in Cx43-interacting protein binding, kinase activity, hemichannel function, gap junctional communication and the underlying signaling pathways affecting cell biological function [12]. LPS induces Cx43 phosphorylation leading to abrogation of gap junction intercellular communication [26]. Similar to the previous study, LPS also induced MtCx43 phosphorylation in HUVECs. However, the ratios of phosphorylated Cx43 in plasma membrane and whole-cell lysate over total Cx43 remained constant after LPS treatment (unpublished data), which was in lines with the precious study [27].

Studies have shown that LPS can activate endothelial cells to promote the expression of nitric oxide (NO), oxygen free radicals, chemokines, cytokines and prostaglandins, which promote cell injury through autocrine or paracrine pathways, and that these injuries were increased by enlarged endothelial cells, exposed basement membrane, and migration of neutrophils and monocytes to endothelium [28, 29]. The damage to vascular endothelial cells induced by LPS and its derivatives has been recognized for a long time and observed in the form of apoptosis, in which oxygen free radicals play an important role [3]. In line with the previous study, our data showed that LPS dramatically increased apoptosis in HUVECs [30]. The apoptosis of vascular endothelial cells not only causes local anticoagulation of endothelial cells, fibrinolysis and decrease of barrier function for preventing lipid deposition, but also induces the release of IL-1α from apoptotic endothelial cells and activates the adjacent endothelial cells to express adhesion molecules and pro-inflammatory cytokines [31]. Endothelial cell apoptosis also resulted in inner membrane injury and thrombosis formation in vascular wall, accelerating the progression of ARDS.

In the present study, we also observed increased intracellular ROS levels in response to LPS stimulation in HUVECs. Oxidative stress induced cell apoptosis when the endogenous antioxidant factors were decreased [32]. ROS plays an important role in the pathogenesis of many inflammatory diseases, including ARDS. Studies reported that stimulation of endothelial cells with endotoxin could lead to an increase in antioxidant enzyme activity and increase ROS activity by upregulating the expression of NAPDH oxidase subunit CYBA [33]. In line with the previous study, some oxidative stress-related factors such as ATOX1 and GSS antioxidant enzymes were decreased, while NAPDH oxidase subunit CYBB was increased in HUVECs after LPS treatment, suggesting that oxidative stress plays an important role in LPS-induced injury in HUVECs. GSS induces an increase in glutathione (GSH) that is important for protection of cells from oxidative stress through free radicals, detoxification of xenobiotics and membrane transport [17]. ATOX1 protects cells against oxidative stress and facilitates the cellular response to oxidized GSH and/or reduced availability of GSH [19]. These data suggest that GSS and ATOX1 may protect cells against LPS-induced oxidative stress through GSH-dependent mechanism. However, the direct effect needs further investigation. GSTZ1, as an antioxidant enzyme, plays a pivotal role in oxidative stress resistance [18] and HMOX1 contains an anti-oxidant response element in the promoter region and is upregulated in response to oxidative stress [20]. However, when challenged by LPS, HMOX1 −/− mice succumb to unfettered oxidative stress and associate with widespread oxidative tissue injury [34], indicating that HMOX1 may protect against LPS-induced oxidative stress. Here, GSTZ1 was increased while HMOX1 was decreased by LPS, which was not fully consistent with the previously described studies. Therefore, the complicated role of these proteins in oxidative stress needs to be further confirmed.

An important finding from our study is that overexpression of Cx43 mimics the effects of LPS on apoptosis and intracellular ROS levels in HUVECs, whereas Gap19 or Cx43 silencing (unpublished data) attenuates these effects, suggesting an involvement of Cx43 in LPS-induced cell injury in HUVECs. Gap19 selectively inhibited plasma membrane Cx43 hemichannels by preventing intramolecular interactions of the Cx43 C-terminus with the cytoplasmic loop, which were essential for Cx43 hemichannel activities, without blocking gap junction channels [15]. Moreover, Gap19 inhibited subsarcolemmal mitochondrial Cx43 hemichannels by mitochondrial potassium and calcium influx and subsequent activation of permeability transition [35, 36]. In the present study, Gap19 significantly reduced the protein level of Cx43 as well as Cx43-mediated effects on oxidative stress-related factors in HUVECs. Studies have shown that MtCx43 may exist in some intracellular signal transduction and paracrine regulation, and may be involved in the regulation of apoptosis [12, 37]. Phosphorylated MtCx43 is common in mitochondria of myocardial and retinal endothelial cells, and MtCx43 channel activity is necessary to maintain mitochondrial morphology [13, 14]. Besides, increased activation of MtCx43 by S-nitrosylation leads to increased ROS and the addition of the non-selective inhibitor Gap26 reduces ROS production [38], suggesting that MtCx43 is a new factor in regulating mitochondrial function. Similarly, inhibition of Cx43 by Gap19 significantly suppressed ROS production and apoptosis in HUVECs. However, the role of Cx43 as well as MtCx43 in mitochondrial physiology during ARDS still needs further investigation.

## Conclusions

In summary, the findings from the present study demonstrate that selective inhibition of Cx43 hemichannels protects HUVECs from LPS-induced apoptosis and this may be via a reduction in oxidative stress production. The protective effects elicited by Cx43 inhibition extend our understanding of the molecular mechanisms underlying the applications of Cx43 for ARDS.

## Data Availability

The datasets generated and/or analyzed during the current study are available from the corresponding author on reasonable request.

## Abbreviations

Annexin V-FITC: Fluorescein isothiocyanate (FITC)-labeled annexin V
ARDS: Acute respiratory distress syndrome
ATOX1: Antioxidant 1
BCA: Bicinchoninic acid
CYBB: Cytochrome b alpha beta
DMEM: Dulbecco’s modified Eagle’s medium
FBS: Fetal bovine serum
GSH: Glutathione
GSS: Glutathione synthetase
GSTZ1: Glutathione S-transferase Zeta 1
HMOX1: Heme oxygenase 1
HUVECs: Human umbilical vein endothelial cells
MtCx43: Mitochondrial connexin 43
PI: Propidium iodide
PMVEC: Pulmonary microvascular endothelial cell
